# Giant parathyroid lipoadenoma case report: A parathyroid phenomenon

**DOI:** 10.1016/j.ijscr.2024.109962

**Published:** 2024-07-02

**Authors:** K. Griffin, K. McKnight, B. Al-Kendi, T. McHale, M.J. Kerin, S. abd Elwahab

**Affiliations:** a Department of Surgery, Galway University Hospitals, Republic of Ireland; b Department of Pathology, Galway University Hospitals, Republic of Ireland

**Keywords:** Parathyroid lipoadenoma, Parathyroid hamartoma, Primary hyperparathyroidism, Hypercalcaemia, Case report

## Abstract

**Introduction:**

Parathyroid lipoadenomas are a rare parathyroid phenomenon and an unusual cause of primary hyperparathyroidism. A difficult diagnosis to make, there are less than 100 cases in the literature since they were first described in 1958, and to our knowledge this is the largest parathyroid lipoadenoma to be reported.

**Presentation of case:**

A minimally-invasive parathyroidectomy with intraoperative parathyroid hormone monitoring was performed in the case of a male with a large neck mass and symptomatic primary hyperparathyroidism. A giant parathyroid lipoadenoma was excised, with an appropriate decrease in intraoperative parathyroid hormone level observed.

**Discussion:**

This lesion poses a challenge to the surgeon, radiologist and pathologist alike and is an important addition to the scant literature available. Clinically it presents similarly to a simple adenoma. The high adipose content of this lesion leads to difficulty localising it on imaging, and the histology study can lead pathologists astray.

**Conclusion:**

We highlight the importance of having the parathyroid lipoadenoma as a differential diagnosis for patients who develop primary hyperparathyroidism.

## Highlights

• PLAs are a rare parathyroid phenomenon, with less than 100 cases reported.
• Potential to mislead multiple specialties involved in patient care.
• Pre-operative imaging often non-diagnostic.
• No agreed histogenesis.
• Adipose volume misleads pathologists, who may provide a report of normal parathyroid tissue.

## 1 Introduction

Primary hyperparathyroidism (PHTP) is reported to be the third most diagnosed endocrine disorder, typically presenting as asymptomatic hypercalcaemia [1]. 80–85 % of primary HPT cases are secondary to a chief cell parathyroid adenoma, understandably so as chief cells constitute the main source of parathyroid hormone (PTH) [2]. Parathyroid lipoadenomas (PLAs) have been defined by the World Health Organisation as a “hamartoma-like benign neoplasm containing both chief cells and prominent stromal elements” [1] that is a well-circumscribed benign lesion [3]. First described in 1962, they are exceptionally rare neoplasms – less than 100 cases are reported as of 2022 [4] with a reported incidence of 0.5–1.6 % [2,5]. There is an equal male-to-female ratio with this tumour, with a reported age range of 41 to 91 years [6]. There is no known association with the MEN syndromes [5,6]. They are difficult to diagnose pre-operatively, as we will detail in this report. Their high adiposity provides challenges to radiologists, surgeons and pathologists alike – distortion of imaging, difficulty making a pre-operative diagnosis, challenging excision, and similarity to normal parathyroid tissue. For this reason, we present the largest known case of a giant parathyroid lipoadenoma, managed in our academic tertiary institution following a chronic history of symptomatic hypercalcaemia, as per the SCARE guidelines [7].

## 2 Presentation of case

**Fig. 1:**
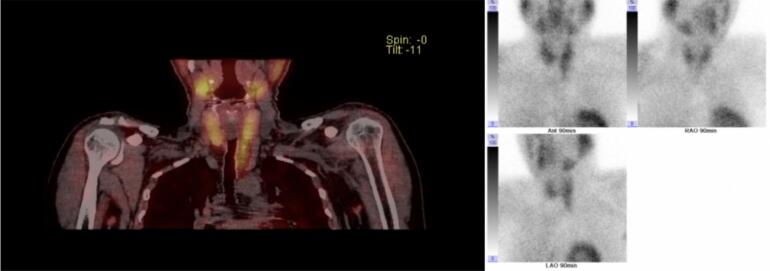
Sestamibi imaging: increased FDG uptake identified near the left inferior pole of the thyroid, extending inferiorly.

A 48 year old male who had been initially investigated in 2019 for a 20 year history of recurrent renal calculi, requiring three lithotripsy and stenting procedures was referred to our tertiary institution's endocrine surgery department in 2023. After this 2019 appointment with an endocrinologist, he was lost to follow-up until he presented to our surgical service complaining of the classic primary hyperparathyroidism pentad: ‘painful bones, kidney stones, abdominal groans, lethargic moans and psychiatric overtones’. He described bone pain unrelated to his daily activities, he had occasional renal colic with a known history of recurrent renal calculi, intermittent, non-specific abdominal pain and lethargy with associated low mood. Apart from being an active smoker with a 30 pack year history, he had a good performance status with no other medical co-morbidities. Our initial investigations revealed a parathyroid hormone level of 187 ng/L (normal range: 10–65 ng/L) and his albumin adjusted calcium level was 2.85 mmol/L having previously been 2.81 mmol/L in 2019 (normal range: 2.20–2.60 mmol/L). He had a normal estimated glomerular filtration rate and Vitamin D level following supplementation by his primary care physician. Per our institution's protocol, neck ultrasound and SPECT-CT sestamibi scintigraphy was performed. The ultrasound showed a slightly enlarged thyroid with no evidence of a parathyroid adenoma. This was discordant with the sestamibi scan which showed increased radioisotope uptake inferior to the lower pole of the left thyroid lobe, appearing to correspond to a fatty mass in the left upper paratracheal region with a presumed adenoma within (Fig. 1).

As the likely causative lesion was localised on sestamibi imaging, we proceeded to perform a minimally-invasive parathyroidectomy under general anaesthesia, led by the Professor of Surgery. Extraneous concurrent treatment was deemed unnecessary in this case. Excision was challenging, as the lesion was large and had extended retrosternally. The soft, fatty nature of the lesion required careful traction and dissection to excise it en-bloc. We employed intraoperative PTH monitoring and the Miami criterion for this case (Table 1), along with nerve monitoring for the safe preservation of the recurrent laryngeal nerve.

**Table 1:** Intraoperative PTH values.

	ioPTH^a^
	ng/L
Pre-operative	416
Pre-excision	796
5 min post-excision	141
10 min post-excision	106

a Intraoperative PTH.

**Fig. 2:**
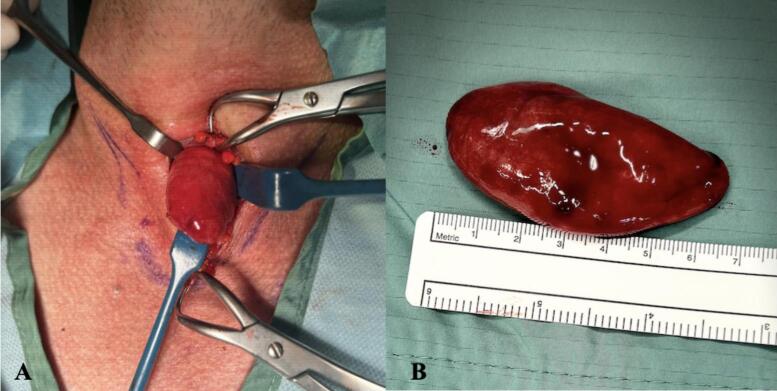
**A.** Pre-excision image of the parathyroid lipoadenoma. **B.** Post-excision image of surgical specimen.

No intraoperative complications were encountered, and he recovered well thereafter. No post-operative complications such as recurrent laryngeal nerve palsy, transient or permanent hypoparathyroidism or wound infection were noted. The 60 × 25 × 18 mm specimen weighed 18 g on excision, with a 9 × 8 × 3 mm well circumscribed firm nodule attached was sent for histology (Fig. 2). This is the largest parathyroid lipoadenoma described in the literature to date.

**Fig. 3:**
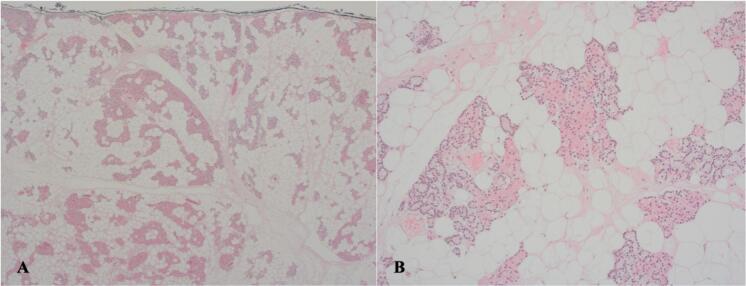
**A.** Lipoadenoma at 4× magnification. **B.** Lipoadenoma at 10× magnification illustrating admixture of chief cells and oxyphil cells intimately associated with adipocytes.

Approximately 60 % of the specimen was sampled, reporting parathyroid tissue containing abundant mature adipose tissue with admixed chief and oxyphil cells in keeping with a parathyroid lipoadenoma (Fig. 3). There was evidence to suggest an underlying malignant process. Two months after intervention, his albumin adjusted calcium level was 2.37 mmol/L and his PTH was 56.8 ng/L and he had complete resolution of his presenting symptoms.

## 3 Discussion

The earliest case similar to this was described in 1958 as a parathyroid hamartoma, due to a lack of functional endocrine activity [8]. Later in 1962, Abul-Haj reported a parathyroid lipoadenoma found to be associated with PHPT [9]. This particular parathyroid lesion has many labels across the literature: adenolipoma, myxoid lipoadenoma, parathyroid hamartoma and parathyroid adenoma with myxoid stroma [8]. There is some debate around what constitutes a parathyroid hamartoma – hamartomas have a more complicated structure, with organoid parenchyma and a greater mix of parathyroid cell types [3]. Hyperfunction is not expected with a hamartoma, which contradicts the basis of PLA causing PHPT.

[New order of paragraphs detailing challenges associated with this case: presentation, surgical, histological].

Patients with PLAs have similar presenting symptoms to those with parathyroid adenomas [3,5]. This, along with the difficulty in identifying them pre-operatively with radiological imaging adds to the diagnostic challenge. The abundance of fatty tissue in the stroma is hyperechoic on ultrasound [10,11], and one case series had a reported ultrasound detection rate of 50 %, while sestamibi scanning had an improved detection of 71 % [12]. Sestamibi imaging has a reported sensitivity of 89–95 %, decreasing in patients who have had repeated surgical intervention [2,13]. The literature is unanimous in supporting the use of sestamibi scanning for pre-operative diagnosis of PLAs, although they can be missed as they can appear to be a space-occupying lesion with low tracer uptake due to the high volume adiposity [13,14]. From the studies of PLAs reviewed for this report, about half were identified as a soft-tissue lesion on ultrasound, with similar numbers being localised on sestamibi imaging.

Curative treatment for PLAs is surgical, requiring pre-operative localisation. Although the ultrasound was of little use, there was FDG uptake indicating localisation to below the left lower lobe of the thyroid on SPECT-CT sestamibi imaging. These images were discussed at the Endocrine Surgery multidisciplinary conference, where the consensus was that there was a likely parathyroid adenoma encased in fatty tissue, and deemed it suitable for excision. Great care is to be taken in order to completely excise the gland. Equally important is intraoperative monitoring of PTH levels, to rule out occult disease in the other parathyroid glands [15]. A reliable predictor of long-term resolution of PHPT is a 50 % decrease of intraoperative PTH level in the 10 min following gland resection [16]. Such was the case with our patient, and this avoided further unnecessary cervical exploration [6].

Our patient did not undergo pre-operative biopsy of the lesion, and so the diagnosis of PLA was made on final histopathological testing. There is no agreed histogenesis of lipoadenoma of the head and neck. Some authors have postulated that the growth stimulus for a gland also acts as a stimulus for adipose cells [9,[17], [18], [19]]. Histopathology examination of these lesions has demonstrated chief cells with no intracellular fat [2], parallel sheets of endoplasmic reticulum and secretory granules around the Golgi apparatus [20], suggestive of hormone production. The specimen we report displayed admixture of chief and oxyphil cells that were intimately associated with the adipose tissue of the excised gland. The amount of adipose tissue in PLAs can be misleading for pathologists, who may incorrectly provide a report of normal parathyroid tissue. It has been suggested that to mitigate this, accurate size and weight of the specimen be provided [2]. A feature of many of the case reports reviewed was that the pathologist did not weigh or measure the specimen before processing, as appearance was consistent with lipomas. An gland is considered enlarged if greater than 6-8 mm and weighing greater than 40-60 mg [2], as in the case of the lesion we describe.

## 4 Conclusion

Our patient presented with signs and symptoms consistent with primary hyperparathyroidism secondary to a hyperfunctioning adenoma. Despite localisation on sestamibi imaging, suspicion of lipoadenoma did not arise until surgical excision. This was further investigated by an astute pathologist who recognised the lipoadenoma pattern of abundant adipose cells interspersed with chief cell and oxyphil cell nodules, nests and cords. This, along with our impression that the gland was hyper-functional, led to the final diagnosis of PLA.

This is an exceedingly rare parathyroid phenomenon, and it is important that cases be added to the literature. While pre-operative imaging has variable rates of localisation success, lipoadenoma should remain a differential for radiologists where there is discordance between imaging modalities, or evidence of increased adiposity surrounding lesions on a background of known hyperfunction of the parathyroid. Excision is more difficult due to the fatty nature of the lesion, and care must be taken to remove it entirely to avoid remnants continuing to cause symptoms for the patient. It is necessary that it be considered as a cause for PHPT, for surgeons, radiologists and pathologists alike, so that patients with primary hyperparathyroidism are identified and managed correctly.

## Informed consent

Informed consent was obtained from the patient to report their case and management.

## Ethical approval

Per the Galway Research Ethics Review Committee, formal ethical approval is not required in the case of a case study of one patient with the proviso that written informed consent has been obtained from the relevant participant. Written informed consent was obtained from the patient for publication and for any accompanying images. A copy of the written consent is available for review by the Editor-in-Chief of this journal on request.

## Funding

The authors did not receive funding to assist with the preparation of this manuscript.

## Author contribution

Conception and design: K Griffin.

Provision of study materials or patients: S abd Elwahab, MJ Kerin, T McHale.

Collection and assembly of data, analysis and interpretation: K Griffin, S abd Elwahab.

Manuscript writing: K Griffin, K McKnight.

Final approval of manuscript: All authors.

## Guarantor

K Griffin.

## Research registration number

Not applicable.

## Declaration of competing interest

The authors declare that there is no conflict of interest regarding the publication of this article.
